# Editorial: Advances in genetics and molecular diagnosis in colorectal, stomach, and pancreatic cancer vol II

**DOI:** 10.3389/fonc.2024.1448183

**Published:** 2024-07-10

**Authors:** Julieta Soarez, Carlos Alberto Vaccaro, Mev Dominguez-Valentin, Walter Hernán Pavicic

**Affiliations:** ^1^ Instituto de Medicina Traslacional e Ingeniería Biomédica (IMTIB) Hospital Italiano de Buenos Aires - Instituto Universitario del Hospital Italiano - Consejo Nacional de Investigaciones Científicas y Técnicas (HIBA-IUHI-CONICET), CABA, Argentina; ^2^ Programa de Cáncer Hereditario (Pro.Can.He.), Hospital Italiano de Buenos Aires (HIBA), CABA, Argentina; ^3^ Department of Tumor Biology, Institute of Cancer Research, The Norwegian Radium Hospital, Oslo, Norway

**Keywords:** gastrointestinal cancer, colorectal cancer, pancreatic cancer, genetic predisposition, hereditary syndrome

Gastrointestinal (GI) cancers encompass a range of malignancies that affect the digestive system. These cancers are among the most prevalent and deadly worldwide, contributing significantly to the global health burden (1). Despite their importance, epidemiological data remain scarce in some regions, impeding effective resource allocation and the implementation of optimal health policies. Moreover, a thorough understanding of the molecular mechanisms behind tumor development is crucial for providing comprehensive oncological care. While advances in omics technologies have significantly deepened our understanding of cancer, additional research is required to fully uncover its molecular and genetic intricacies, especially in relation to diagnosis.

In this Volume II of our Research Topic, we aim to expand upon the concepts introduced in the first volume (2). The article by Olkinuora et al. brings novel insight into the genetic and epigenetic factors involved in the tumorigenesis of Lynch Syndrome (LS), thereby extending our initial focus on genetic predisposition in GI cancers. Additionally, the six articles that complete this series delves deeper into various aspects of GI cancer epidemiology, the necessity of implementing molecular tools for accurate diagnosis, and the latest advances in biomarkers related to tumor development. Together, these contributions enhance our understanding and clarity in this field.

The incidence of GI cancers is not adequately documented in many developing countries, and even in some developed countries. In this context, the articles by Long et al. on CRC in Europe and by Xiang et al. on pancreatic cancer in Asia, reported temporal trends, and geographical and gender differences of the burden of these cancers. Both studies analyzed data spanning three decades (1990-2019), obtained from the Global Burden of Diseases database. As a result, they observed that higher burden was found in men than women and the incidence and burden increased according to the age. Furthermore, disability-adjusted life years (DALYs) related to these cancers have increased, although the age-standardized DALY rate has decreased for CRC.

Regarding the disease burden, significant differences have been described according to the region. The highest rate of pancreatic cancer was reported in the Central Asia region, while the lowest were found in the Southern Asia region. Similarly, the burden of CRC showed significant differences among European countries: Germany having the highest rate, while Andorra had the lowest. The findings presented here offer essential insights for designing robust and context-specific prevention and treatment strategies.

Lynch syndrome is an autosomal dominant multi-organ cancer syndrome with a high lifetime risk of cancer and is the most common hereditary form of hereditary CRC(3). The cumulative number of gastric polyps in LS typically does not exceed ten, and their removal through regular screening significantly reduces cancer risk. However, atypical phenotypes can complicate initial diagnosis, potentially leading to suboptimal treatment. Olkinuora et al. investigated the molecular characteristics and causes of higher number of adenomas in LS patients. They found that frequent variants in specific -critical- regions of the *RNF43* gene, combined with the SBS96 mutational signature and a higher tendency for DNA methylation, could influence tumor multiplicity in this syndrome.

In the context of cancer heterogeneity, precise diagnosis can be delayed when molecular or clinical features overlap with those of other diseases. Early identification of these atypical cancers is crucial to improving patient treatment and prognosis. Accurate diagnosis of patients with NTRK-rearranged spindle cell neoplasms (NTRK-RSCNs) is often delayed due to molecular similarities and overlap with other diseases, such gastrointestinal stromal tumors (GIST). This is particularly true in the absence of thorough morphological and immunohistochemical characterization. Cao et al. reported an adult patient case diagnosed with malignant NTRK-RSCN in the pelvic region. Immunohistochemical tests revealed the presence and absence of certain markers (e.g., vimentin and Ki67, and Desmin and DOG1, respectively), while molecular analysis showed a *TPM3-NTRK1* fusion and the absence of mutations in c-KIT and PDGFRα. Early detection of these cases is essential to personalize and optimize treatment strategies, thus improving clinical outcomes and patient quality of life.

The analysis of molecular pathways in GI cancers and other types of tumors reveals significant complexity in tumorigenesis and progression. Several authors have addressed this topic through reviews. Zhao and Xu examined the variability in *PITX1* gene expression in various malignancies, exploring its possible dual role as an oncogene and tumor suppressor by interacting with key genes such as *P53* and *RASAL1*. They also highlighted the epigenetic regulation of *PITX1*, noting potential therapeutic avenues yet to be explored. Similarly, Scarini et al. evaluated how Eph/ephrin interaction alternates between tumor suppressive and promoting functions, complicating its role in CRC, and underscoring the need for further research to tailor therapies to each patient. On the other hand, Xie et al. summarized the most notable loss of imprinting (LOI) genes in cancer. These include 13 individual genes (including two gene clusters) and 12 combined forms of multigene LOI tests in 16 imprinted genes. The combination of LOI forms has proven effective in cancer diagnosis, using detection methods such as biallelic expression and methylation of differentially methylated regions.

These studies underscore the importance of comprehensive research in advancing effective and personalized therapeutic strategies within the field of precision oncology. The diverse collection of articles reinforces the notion that gaining deeper insights into the molecular and genetic factors associated with the development of GI cancers can significantly enhance the translation of basic research into practical clinical services. By advocating personalized medicine, the effectiveness of treatment against these malignant neoplasms that affect the gastrointestinal tract can be improved.

## Author contributions

JS: Writing – original draft, Writing – review & editing. CV: Writing – review & editing. MD: Writing – review & editing. WP: Writing – original draft, Writing – review & editing.
